# Responsiveness of the individual work performance questionnaire

**DOI:** 10.1186/1471-2458-14-513

**Published:** 2014-05-27

**Authors:** Linda Koopmans, Jennifer K Coffeng, Claire M Bernaards, Cécile RL Boot, Vincent H Hildebrandt, Henrica CW de Vet, Allard J van der Beek

**Affiliations:** 1 Body@Work, Research Center for Physical Activity, Work and Health, TNO-VU University Medical Center, Amsterdam, The Netherlands; 2 Expertise Center Life Style, TNO, PO Box 2215, Leiden, The Netherlands; 3 Department of Epidemiology and Biostatistics, EMGO + Institute for Health and Care Research, VU University Medical Center, Amsterdam, The Netherlands; 4 Department of Public and Occupational Health, EMGO + Institute for Health and Care Research, VU University Medical Center, Amsterdam, The Netherlands

## Abstract

**Background:**

Individual work performance is an important outcome measure in studies in the workplace. Nevertheless, its conceptualization and measurement has proven challenging. To overcome limitations of existing scales, the Individual Work Performance Questionnaire (IWPQ) was recently developed. The aim of the current study was to gain insight into the responsiveness of the IWPQ.

**Methods:**

Data were used from the Be Active & Relax randomized controlled trial. The aim of the trial was to investigate the effectiveness of an intervention to stimulate physical activity and relaxation of office workers, on need for recovery. Individual work performance was a secondary outcome measure of the trial. In total, 39 hypotheses were formulated concerning correlations between changes on the IWPQ scales and changes on similar constructs (e.g., presenteeism) and distinct constructs (e.g., need for recovery) used in the trial.

**Results:**

260 Participants completed the IWPQ at both baseline and 12 months of follow-up. For the IWPQ scales, 23%, 15%, and 38%, respectively, of the hypotheses could be confirmed. In general, the correlations between change scores were weaker than expected. Nevertheless, at least 85% of the correlations were in the expected direction.

**Conclusions:**

Based on results of the current study, no firm conclusions can be drawn about the responsiveness of the IWPQ. Several reasons may account for the weaker than expected correlations. Future research on the IWPQ’s responsiveness should be conducted, preferably in other populations and intervention studies, where greater changes over time can be expected.

## Background

Individual work performance, defined as "*employee behaviours or actions that are relevant to the goals of the organization*" [1], is an important outcome measure in studies in the workplace. The conceptualization of IWP has a long history, and many frameworks have been proposed to describe the construct domain of IWP [e.g., 1–3]. In the field of occupational health, for example, the main focus is on sickness absenteeism or presenteeism, i.e., work absence or losses in IWP due to health impairments. In the field of work and organizational psychology, traditionally, the main focus of the IWP construct has been on task performance, which can be defined as "the proficiency with which individuals perform the core substantive or technical tasks central to his or her job" [1]. It is now generally agreed upon that, in addition to task performance, the IWP domain consists of contextual performance and counterproductive work behaviour [2-4]. Contextual performance can be defined as "behaviours that support the organizational, social and psychological environment in which the technical core must function" [5]. Counterproductive work behaviour can be defined as "behaviour that harms the well-being of the organization" [3].

Considering the diversity in conceptual frameworks of IWP, it is not surprising that numerous instruments have been developed to measure (aspects of) IWP. Numerous and diverse behaviours, actions, or results are being applied as indicators of IWP [6]. In occupational health, numerous instruments have been developed to measure sickness absenteeism or presenteeism, such as the Work Productivity And Impairment Questionnaire [7], Work Limitations Questionnaire [8], and the WHO Health and Performance Questionnaire [9]. Also, work and organizational psychologists have developed numerous scales to measure task performance e.g., [10], contextual performance e.g., [11], or counterproductive work behaviour e.g., [12].

However, all these scales show several limitations. Most strikingly, none of them measure all of the relevant dimensions of IWP together. Thus, they do not measure the full range of IWP. Also, scales measuring different dimensions can include items overlapping in content (*antithetical items*), creating unjust overlap between these scales [13]. As a result, the content validity of these scales can be questioned. Furthermore, none of the scales appear suitable for generic use. The scales were developed for specific populations, such as employees with health problems e.g., [7-9], or they were developed and refined based on employees with a specific occupation e.g., [10-12].

The lack of consensus on how to conceptualize and measure IWP is undesirable, because valid measurement is a prerequisite for accurately establishing, for example, predictors of IWP, or effectiveness of interventions to improve IWP. To overcome the aforementioned limitations, the Individual Work Performance Questionnaire (IWPQ) was recently developed [14,15]. The IWPQ is based on a three-dimensional conceptual framework of IWP, which was developed after a systematic review of the occupational health, psychology, and management literature [4]. This framework includes the aforementioned dimensions of task performance, contextual performance, and counterproductive work behaviour. The IWPQ is a generic instrument, thus, it is suitable for workers in all types of occupations (i.e., blue, pink, and white collar workers) and workers with and without health complaints.

An important purpose of the IWPQ is to assess changes in IWP. For example, we may want to examine fluctuations in IWP over time (e.g., due to age), follow the effects of negative factors on IWP over time (e.g., health problems), or identify successful methods to improve IWP (e.g., intervention studies). In order to do this, the IWPQ must be responsive to changes over time. Responsiveness can be defined as "the ability of an instrument to detect change over time in the construct to be measured" [16]. There is a lot of confusion about the concept of responsiveness, and many different definitions and measures have been proposed over the past decades [17]. For example, the definition of responsiveness has been clouded by a lack of distinction between cross-sectional and longitudinal validity. Secondly, it has been clouded by a lack of distinction between the effect of an intervention, and the correlation of changes in the instrument with changes in other instruments [18]. Also, responsiveness is often examined using inappropriate outcome measures, such as effect sizes or standardized response mean [17]. Perhaps as a result of this unclarity, responsiveness is a seldom examined issue. In the current study, we focus on the validity of a *change score*, which is estimated on the basis of two or more measurement points [17]. The aim of the current study was to gain insight into the responsiveness of the IWPQ.

## Methods

### Participants

Data were used from the Be Active & Relax "Vitality in Practice" (VIP) randomized controlled trial [19]. The aim of the Be Active & Relax trial was to investigate the effectiveness of an intervention to stimulate physical activity and relaxation of office workers, on need for recovery. In September 2011, an invitation was sent to 1,182 office employees of a financial service provider in The Netherlands, to participate in the project. A total of 412 employees (response: 35%) from 19 departments completed the baseline questionnaire and signed the informed consent form, and were included in the trial.

The trial included a 2x2 factorial design with four research arms. The four arms consisted of a combined social and physical environmental intervention, a social environmental intervention only, a physical environmental intervention only and a control group. The social environmental intervention consisted of Group Motivational Interviewing (GMI). GMI is a counseling style that focuses on behavioural change in groups and is derived from Motivational Interviewing at the individual level. GMI was delivered by the teamleaders of the departments. The teamleaders received a two-day training by a GMI-professional. The trained teamleaders then gave three GMI-sessions of 90 minutes each to their own team, within a period of six weeks (i.e. three weeks between each session). Two months after the final session, a booster session was given by the teamleader. All sessions took place during work hours. The main aim of these sessions was to stimulate physical activity and relaxation. For the physical environmental intervention, at six departments, several VIP ("Vitality in Practice") zones were created: (1) the VIP Coffee Corner Zone (4 elements) – the coffee corner was modified by adding a bar table, bar chairs, a large plant and a giant wall poster (a poster visualizing a relaxing environment, e.g. wood, water and mountains); (2) the VIP Open Office Zone (2 elements) – the office was modified by introducing exercise balls and curtains to divide desks in order to reduce background noise; (3) the VIP Meeting Zone (2 elements) – conference rooms were modified by placing a standing table and a giant wall poster (a poster visualizing a relaxing environment, e.g. wood, water and mountains); and (4) the VIP Hall Zone (3 elements) - table tennis tables were placed and lounge chairs were introduced in the hall for informal meetings. In addition, footsteps were placed on the floor in the entrance hall to promote stair walking. By means of stimulating physical activity and relaxation, work-related outcomes (e.g., sickness absenteeism, work engagement and individual work performance) were expected to improve for the intervention groups compared to the control group. For the purpose of the current study, data of all four groups were taken together. This study was approved by the Medical Ethics Committee of the VU University Medical Center, Amsterdam, The Netherlands. Full details of the design of the Be Active & Relax trial have been reported elsewhere [19].

### Measures

As examining the responsiveness of the IWPQ was not a main aim of the Be Active & Relax trial, measurement instruments were included that represented important outcomes in the trial. Measurements took place at baseline (T0), and at 6 months (T1) and 12 months (T2) follow-up. Only the measurements at baseline and at 12 months (T2) were used to assess responsiveness of the IWPQ.

**Individual work performance** was measured using the Individual Work Performance Questionnaire (IWPQ) [14,15]. The IWPQ consists of 18 questions in three scales: task performance (5 items), contextual performance (8 items), and counterproductive work behaviour (5 items). The IWPQ has a recall period of 3 months and a rating scale from 0 ("*seldom")* to 4 (*"always")* for task and contextual performance, and 0 (*"never")* to 4 (*"often")* for counterproductive work behaviour. For the IWPQ subscales, a mean score is calculated by adding the item scores, and dividing their sum by the number of items in the subscale. Hence, the IWPQ yields three subscale scores that range between 0 and 4, with higher scores reflecting higher task and contextual performance, and higher counterproductive work behaviour. The psychometric properties of the IWPQ have been tested and results indicated good to excellent internal consistency for task performance (α = 0.78), contextual performance (α = 0.85) and counterproductive work behaviour (α = 0.79). The IWPQ has shown good face and structural validity [6,14,15], as well as sufficient convergent validity and good discriminative validity [20].

**Presenteeism**, which can be defined as "decreased on-the-job performance due to the presence of health problems" [21], was assessed through self-report with the World Health Organization Health and Work Performance Questionnaire (WHO-HPQ) [9]. Presenteeism was assessed by asking participants to rate their actual performance in relation to possible performance. The score represents percentage of performance, and has a lower bound of 0 (*total lack of performance*) and an upper bound of 100 (*top performance*). The reliability and validity of the HPQ was examined for several occupations, and showed good convergent validity. However, poor validity was found for white collar workers [9,22].

**Job satisfaction** was assessed using one overall question ("*Overall, how satisfied are you with your job?*") on a rating scale from 1 ("*highly dissatisfied*") to 5 ("*very satisfied*"). A single-item measure of job satisfaction has been found to correlate highly with job satisfaction scales, and was therefore considered valid [23,24].

**Work engagement** was measured using the Utrecht Work Engagement Scale (UWES) [25]. The UWES consists of three scales (vigour, dedication, and absorption), and a total of 17 items assessed on a rating scale from 1 ("*never*") to 7 ("*always*"). The total score was calculated by adding the means of each scale, and dividing the sum by three. The psychometric properties of this questionnaire have been tested and results indicated an acceptable reliability of vigour (α = 0.68-0.80), dedication (α = 0.91) absorption (α = 0.73-0.75), and the total score (α = 0.93), as well as acceptable convergent validity [25].

**Work ability** was assessed using one question ("*How do you rate your current work ability compared to lifetime best?*") from the Work Ability Index (WAI) [26], on a rating scale from 1 ("*completely unable to work*") to 10 ("*at its best*"). The single-item question is very strongly associated with the total WAI, and has shown good predictive validity [27].

**Performance rating by the manager** was assessed by asking one self-report question ("*How would your manager rate your overall job performance, compared to colleagues in a similar job?")* on a rating scale from 1 ("*much worse*") to 5 ("*much better*"). This question was adapted from the WHO-HPQ [9] presenteeism question, and previously used in The Netherlands Working Conditions Survey [28]. The reliability and validity of this question is unknown.

**Self-rated work quality and quantity** were assessed using one question each ("*How do you rate the quality of your own work?" and "How do you rate the quantity of your own work?")* on a rating scale from 1 ("*insufficient*") to 5 ("*excellent*"). The reliability and validity of these questions is unknown.

**Need for recovery** (NFR) was assessed using the Need for Recovery after Work scale [29]. This Dutch version of the Questionnaire on the Experience and Evaluation of Work (Dutch abbreviation: VBBA) consists of eleven dichotomous items (yes/no), representing short-term effects of a day at work. The NFR score is a percentage score (0 to 100) of positive answers of those providing data for at least 8 of the 11 items. The Need for Recovery after Work scale has shown good reliability (α = 0.86-0.88), construct validity, and responsiveness in The Netherlands [29-31].

**Physical activity** was assessed using the Short Questionnaire to Assess Health Enhancing Physical Activity (SQUASH) [32]. Duration and intensity of active commuting, leisure time activities, sport activities, household activities, and physical activities at work (standing and walking), were assessed. For each domain, employees were asked to report the frequency (i.e., times per week), duration of activities (i.e., in minutes), and self-reported intensity (i.e., light, moderate or vigorous). Total scores for minutes per week spent on light, moderate, and vigorous physical activities were calculated. The SQUASH scores have shown reasonable reproducibility (r = 0.57-0.58) and validity against accelerometry (r = 0.45-0.67), which is comparable to other physical activity questionnaires [32,33].

**General health** and **vitality** were measured using the Dutch version of the Rand-36 [34]. General health was measured by asking workers to indicate how they perceived their general health, on a rating scale from 1 ("*poor*") to 5 ("*excellent*"). Vitality was measured with a scale of 5 items, asking workers to indicate how often they felt full of life, worn out, tired and full of energy, on a rating scale from 1 ("*never*") to 6 ("*always*"). This scale was transformed to a 0–100 score, with higher scores indicating higher vitality. The Dutch version of the Rand-36 has shown good reliability for the vitality scale (α = 0.82) and had reasonable construct validity [34].

**Exhaustion** was measured using the OLdenburg Burnout Inventory (OLBI) [35]. The OLBI consists of eight items on a 4-point scale ranging from 1 ("*totally disagree*") to 4 ("*totally agree*"). A mean score was calculated. The OLBI has shown good reliability (α = 0.80-0.85) and reasonable convergent and discriminant validity in different occupational groups [35,36].

**Sickness absenteeism** data were retrieved from company records, for the year prior to the intervention (i.e. baseline), and for the year of the intervention (i.e., 12 month follow-up). The score represents the number of workdays absent per year.

### Correlations between change scores

A construct approach of responsiveness testing [17] was applied in the current study, which means that hypotheses were formulated concerning relationships between changes on the IWPQ and changes on other instruments used in the Be Active & Relax trial. Based on the literature, hypotheses concerning the relationships between changes on the IWPQ scales and changes on other instruments were formulated. In line with Cohen [37], we interpreted a correlation coefficient over 0.50 as strong, 0.30 to 0.50 as moderate, 0.10 to 0.30 as weak, and below 0.10 as no relation between constructs at all. When moderate correlations were expected, based on literature, we classified these constructs as similar constructs (e.g., presenteeism). When weak correlations or no correlations were expected, we classified these constructs as distinct constructs (e.g., need for recovery). Based on the literature, expectations were formulated per IWPQ scale, resulting in a total of 39 hypotheses (3 IWPQ scales × 13 constructs). If positive correlations were expected for task and contextual performance, negative correlations were expected for counterproductive work behaviour, and vice versa.

#### Hypotheses with similar constructs

The first 21 hypotheses (3 IWPQ scales × 7 constructs) concern relationships of the IWPQ scales with similar constructs. These constructs were classified as similar constructs, because these constructs were theoretically expected to correlate moderately with work performance, or were found to correlate moderately with work performance in previous research. For example, in a review by Judge et al. [38], the correlation between overall job satisfaction and work performance was estimated to be 0.30. Therefore, the change in the IWPQ task and contextual performance scale was expected to correlate moderately positive (0.30–0.50) with the change in presenteeism [20], job satisfaction e.g., [38], work engagement e.g., [39], work ability e.g., [40], performance rating by the manager [41], work quality, and work quantity. The change in the IWPQ counterproductive work behaviour scale was expected to correlate moderately negative (-0.50–-0.30) with the change in presenteeism [20], job satisfaction e.g., [38], work engagement e.g., [39], and work ability e.g., [40]. Based on literature, the change in the IWPQ counterproductive work behaviour scale was expected to correlate weakly or not at all (-0.20–0.20) with the change in performance rating by the manager, work quality, and work quantity [13].

#### Hypotheses with distinct constructs

The last 18 hypotheses (3 IWPQ scales × 6 constructs) concern relationships of the IWPQ scales with distinct constructs. These constructs were classified as distinct constructs, because these constructs were theoretically expected to correlate weakly or not at all with work performance, or were found to correlate weakly or not at all with work performance in previous research. For example, it was found that absenteeism is not strongly related to work performance [42,43]. Therefore, the change in the IWPQ task and contextual performance scale was expected to correlate weakly positive (0.20–0.30), and the change in the IWPQ counterproductive work behaviour scale weakly negative (-0.30–-0.20), with the change in need for recovery e.g., [36,44], physical activity e.g., [45], general health e.g., [21,46], vitality e.g., [47], and exhaustion e.g., [48]. Finally, the change in each IWPQ scale was expected to correlate weakly or not at all (-0.20–0.20) with the change in sickness absenteeism [42,43].

### Data analysis

Pearson correlations between the change scores of each IWPQ scale and the change scores on the other constructs were calculated for the change scores from baseline (T0) to 12 months (T2). To examine the magnitude of the changes, respondents were divided in three groups: those who decreased at least one point on a construct, those who increased at least one point on a construct, and those who changed less than one point on a construct. For the decrease and increase groups, the mean change and SD of change were calculated for the IWPQ scales. Only participants who completed the IWPQ at both T0 and T2 were included in the data analysis. Analyses were conducted in SPSS 20.0 [49].

## Results

### Descriptive statistics of the participants

Of the 412 participants in the Be Active & Relax trial, 260 participants (63%) completed the IWPQ at both baseline and 12 months. The main reasons for loss-to-follow-up were changing job to a different employer and lack of motivation. At baseline (n = 260), participants had a mean age of 43.2 years (SD = 9.9), worked 36 hours per week (SD = 5.1), most were male (63%), and most were highly educated (79%). On average, participants rated their general health as good (M = 3.35, SD = 0.85, on a 5-point scale), and had an average BMI of 25.11 (SD = 4.07).

### Descriptive statistics of the IWPQ scales and the other constructs

Table 1 presents the mean scores and standard deviations (SD) on the IWPQ scales and the other constructs at baseline (T0) and 12 months (T2). It also reports the mean and standard deviation (SD_change_) of the change scores on the IWPQ scales and the other constructs from T0 to T2.

**Table 1:** **Mean scores (and SD) and mean change scores (and SD**_**change**_**) on the IWPQ scales and the similar/distinct constructs at baseline (T0) and 12 months (T2)**

	**T0**	**T2**	**Change score**
	**(baseline)**	**(12 months)**	**T2-T0**
	**n = 412**	**n = 260**	**n = 260**
	**Mean (SD)**	**Mean (SD)**	**Mean (SD**_**change**_**)**
**IWPQ (0–4)**			
Task performance	2.46 (0.68)	2.63 (0.66)	0.17 (0.70)
Contextual performance	2.34 (0.71)	2.39 (0.79)	0.04 (0.69)
Counterproductive work behaviour	1.23 (0.65)	1.16 (0.66)	-0.07 (0.64)
*Similar constructs*			
Presenteeism (0–100)	76.58 (8.76)	75.87 (10.62)	-0.79 (11.51)
Job satisfaction (1–5)	3.96 (0.73)	3.85 (0.75)	-0.11 (0.80)
Work engagement (1–7)	4.91 (0.85)	4.84 (0.93)	-0.07 (0.71)
Work ability (1–10)	7.79 (1.42)	7.70 (1.57)	-0.08 (1.56)
Performance rating by the manager (1–5)	3.41 (0.81)	3.46 (0.81)	0.06 (0.81)
Self-rated work quality (1–5)	3.83 (0.79)	3.63 (0.87)	-0.20 (0.95)
Self-rated work quantity (1–5)	3.87 (0.83)	3.74 (0.92)	-0.12 (0.95)
*Distinct constructs*			
Need for recovery (0–100)	32.20 (29.26)	27.78 (28.71)	-2.40 (23.70)
Physical activity (*min/week*)			
- Light	1810.10 (1363.68)	1603.23 (1618.94)	-199.40 (1785.64)
- Moderate	281.81 (254.19)	350.94 (633.98)	72.66 (629.00)
- Vigorous	83.53 (160.15)	99.79 (272.90)	9.40 (266.15)
General health (1–5)	3.35 (0.85)	3.37 (0.84)	0.79 (1.53)
Vitality (0–100)	64.08 (18.84)	65.72 (17.97)	1.87 (15.17)
Exhaustion (1–4)	2.15 (0.48)	2.15 (0.46)	0.04 (0.40)
Sickness absenteeism (*workdays absent per year*)	7.55 (21.81)	7.37 (20.91)	0.55 (25.03)

### Correlations between change scores

Table 2 presents the expected and observed correlations between the change scores of the IWPQ scales and the change scores of the other constructs. For task performance, 85% of the correlations were in the expected direction, and for contextual performance and counterproductive work behaviour, 92% of the correlations were in the expected direction. However, in many cases, the correlations were weaker than expected.

**Table 2:** Pearson correlations (E = expected, O = observed) between change scores of the IWPQ scales and similar/distinct constructs (n = 260)

	**IWPQ scale**		
	**Task performance**	**Contextual performance**	**Counterproductive work behaviour**
*Similar constructs*			
Presenteeism	E: 0.30 – 0.50	E: 0.30 – 0.50	E: -0.50 – -0.30
	O: 0.18	O: 0.22	O: -0.11
Job satisfaction	E: 0.30 – 0.50	E: 0.30 – 0.50	E: -0.50 – -0.30
	O: 0.12	O: 0.17	O: -0.24
Work engagement	E: 0.30 – 0.50	E: 0.30 – 0.50	E: -0.50 – -0.30
	O: 0.19	O: 0.29	O: -0.23
Work ability	E: 0.30 – 0.50	E: 0.30 – 0.50	E: -0.50 – -0.30
	O: 0.16	O: 0.26	O: -0.23
Performance rating by the manager	E: 0.30 – 0.50	E: 0.30 – 0.50	E: -0.20 – 0.20
	O: 0.16	O: 0.22	O: -0.02*
Work quality	E: 0.30 – 0.50	E: 0.30 – 0.50	E: -0.20 – 0.20
	O: 0.20	O: 0.18	O: -0.06*
Work quantity	E: 0.30 – 0.50	E: 0.30 – 0.50	E: -0.20 – 0.20
	O: 0.11	O: 0.19	O: 0.02*
*Distinct constructs*			
Need for recovery	E: -0.30 – -0.20	E: -0.30 – -0.20	E: 0.20 – 0.30
	O: -0.15	O: -0.11	O: 0.16
Physical activity	E: 0.20 – 0.30	E: 0.20 – 0.30	E: -0.30 – -0.20
- Light	O: -0.09	O: -0.04	O: -0.07
- Moderate	O: 0.03	O: 0.03	O: -0.07
- Vigorous	O: -0.05	O: 0.00	O: -0.04
General health	E: 0.20 – 0.30	E: 0.20 – 0.30	E: -0.30 – -0.20
	O: -0.07	O: 0.08	O: 0.02
Vitality	E: 0.20 – 0.30	E: 0.20 – 0.30	E: -0.30 – -0.20
	O: 0.23*	O: 0.29*	O: -0.03
Exhaustion	: -0.30 – -0.20	E: -0.30 – -0.20	E: 0.20 – 0.30
	O: -0.23*	O: -0.13	O: 0.23*
Sickness absenteeism	E: -0.20 – 0.20	E: -0.20 – 0.20	E: -0.20 – 0.20
	O: -0.14*	O: -0.08*	O: -0.09*
**Hypotheses:**			
Confirmed	23%	15%	38%
In the right direction	85%	92%	92%

Note: E = expected correlation, O = observed correlation. * = Confirmed hypothesis.

For the task performance scale, 3 out of 13 (23%) hypotheses were fully confirmed. As expected, the change in task performance correlated moderately positive with the changes in vitality (*r* = 0.23), moderately negatively with the change in exhaustion (*r* = -0.23), and weakly negative with the change in absenteeism (*r* = -0.14).

For the contextual performance scale, 2 out of 13 (15%) hypotheses were fully confirmed. As expected, the change in contextual performance correlated moderately positive with the change in vitality (*r* = 0.29), and weakly negative with the change in absenteeism (*r* = -0.08). Furthermore, the correlation between the change in contextual performance and the changes in most of the similar constructs (e.g., presenteeism, work engagement, work ability) approached the 0.30 correlation strength.

For the counterproductive work behaviour scale, 5 out of 13 (38%) hypotheses were fully confirmed. As expected, the change in counterproductive work behaviour correlated weakly with the changes in rating by the manager (*r* = -0.02), work quality (*r* = -0.06), work quantity (*r* = 0.02), and absenteeism (*r* = -0.09), and moderately positive with the change in exhaustion (*r* = 0.23).

In sum, 23%, 15%, and 38% of the hypotheses could be confirmed for the IWPQ task performance, contextual performance, and counterproductive work behaviour scales, respectively. As hypothesized, the correlations of the IWPQ scales were slightly stronger with similar constructs than with distinct constructs, on average. However, in general, the correlations between change scores were weaker than expected. Nevertheless, most of the correlations (at least 85%) were in the expected direction. Exceptions were the correlations between the change scores of task performance and light and intense physical activity (r = -0.09 and -0.05, respectively), task performance and general health (r = -0.07), contextual performance and light physical activity (r = -0.04), and counterproductive work behaviour and general health (r = 0.02).

### Magnitude of change

Table 3 presents the number of participants that respectively decreased or increased at least one point on a construct from T0 to T2, and their corresponding mean change and standard deviation of change on the IWPQ scales. For example, of the 260 participants, 111 participants reported a decreased need for recovery of at least one point. On average this group showed an increase in task performance (M_change_ = 0.27, SD_change_ = 0.65), an increase of contextual performance (M_change_ = 0.12, SD_change_ = 0.65), and a decrease in counterproductive work behaviour (M_change_ = -0.11, SD_change_ = 0.63). A total of 91 participants reported an increased need for recovery. On average this group showed a slight increase in task performance (M_change_ = 0.10, SD_change_ = 0.76), a slight decrease in contextual performance (M_change_ = -0.07, SD_change_ = 0.82), and a slight increase in counterproductive work behaviour (M_change_ = 0.02, SD_change_ = 0.70).

**Table 3:** **Number of participants (n) that respectively decreased or increased at least one point on a construct from T0 to T2, and their corresponding mean change and standard deviation of mean change (SD**_**change**_**) on the IWPQ scales**

			**IWPQ**		
			**Task performance**	**Contextual performance**	**Counterproductive work behaviour**
			**Mean change**	**Mean change**	**Mean change**
	**T0-T2**	**n**	**(SD**_**change**_**)**	**(SD**_**change**_**)**	**(SD**_**change**_**)**
*Similar constructs*					
Presenteeism	Decreased	62	-0.07 (0.83)	-0.16 (0.73)	0.09 (0.67)
	Increased	61	0.18 (0.57)	0.23 (0.71)	-0.08 (0.71)
Job satisfaction	Decreased	56	0.07 (0.84)	-0.17 (0.87)	0.21 (0.81)
	Increased	36	0.24 (0.66)	0.16 (0.62)	-0.42 (0.58)
Work engagement	Decreased	19	-0.16 (1.09)	-0.30 (0.79)	0.26 (0.77)
	Increased	12	0.27 (0.94)	0.21 (0.73)	-0.23 (0.62)
Work ability	Decreased	77	0.04 (0.71)	-0.17 (0.70)	-0.07 (0.69)
	Increased	78	0.32 (0.75)	0.24 (0.71)	-0.15 (0.66)
Performance rating by the manager	Decreased	39	-0.07 (0.62)	-0.19 (0.63)	-0.08 (0.62)
	Increased	52	0.20 (0.81)	0.19 (0.70)	-0.08 (0.69)
Self-rated work quality	Decreased	78	-0.01 (0.80)	-0.14 (0.74)	-0.04 (0.74)
	Increased	43	0.33 (0.67)	0.23 (0.73)	-0.07 (0.61)
Self-rated work quantity	Decreased	64	0.10 (0.79)	-0.09 (0.80)	-0.14 (0.72)
	Increased	52	0.24 (0.57)	0.15 (0.65)	-0.11 (0.52)
*Distinct constructs*					
Need for recovery	Decreased	111	0.27 (0.65)	0.12 (0.65)	-0.11 (0.63)
	Increased	91	0.10 (0.76)	-0.07 (0.82)	0.02 (0.70)
Physical activity	Decreased	79	0.23 (0.77)	0.09 (0.70)	0.03 (0.62)
	Increased	181	0.15 (0.66)	0.02 (0.68)	-0.11 (0.64)
General health	Decreased	36	0.44 (0.74)	-0.07 (0.77)	-0.26 (0.70)
	Increased	114	0.15 (0.63)	0.09 (0.68)	-0.09 (0.58)
Vitality	Decreased	95	0.03 (0.75)	-0.16 (0.68)	-0.02 (0.65)
	Increased	108	0.30 (0.67)	0.18 (0.66)	-0.10 (0.66)
Exhaustion	Decreased	4	0.30 (0.99)	0.49 (0.43)	-0.70 (1.09)
	Increased	7	-0.34 (0.91)	0.29 (0.74)	0.43 (0.76)
Absenteeism	Decreased	84	0.25 (0.71)	0.08 (0.68)	-0.05 (0.67)
	Increased	73	0.06 (0.66)	-0.08 (0.70)	-0.13 (0.62)

Similarly, for example, a total of 81 participants reported a decrease in presenteeism of at least one point. On average this group showed a slight decrease in task performance (M_change_ = -0.07, SD_change_ = 0.83), a decrease in contextual performance (M_change_ = -0.16, SD_change_ = 0.73), and a slight increase in counterproductive work behaviour (M_change_ = 0.09, SD_change_ =0.67). A total of 61 participants reported an increase in presenteeism. On average this group showed an increase in task performance (M_change_ = 0.18, SD _change_ = 0.57) an increase in contextual performance (M_change_ = 0.23, SD_change_ = 0.71), and a slight decrease in counterproductive work behaviour (M_change_ = -0.08, SD_change_ = 0.71). As can be observed in the above examples, most of the time, a decrease or increase in a construct was associated with a corresponding decrease or increase in the IWPQ scales.

## Discussion

The aim of the current study was to examine the responsiveness of the IWPQ, i.e., the ability of the IWPQ to detect change over time. A total of 39 hypotheses were formulated concerning the relationships between changes on the IWPQ and changes on similar constructs (e.g., presenteeism) and distinct constructs (e.g., need for recovery) used in the Be Active & Relax trial. Although most of the correlations between change scores were in the expected direction, most were weaker than expected. Several reasons may account for this.

First, the IWPQ questions may not be sensitive enough to pick up changes in IWP over time. Also, it is hard to say how a change from answer categories "regularly" to "often" can be achieved. What needs to be done to accomplish a change from "regularly" to "often," e.g., in keeping your work results in mind? And what does this change mean? In sum, the questions of the IWPQ scales may lack discriminative ability. However, in the developmental phase of the IWPQ scales, Rasch analysis [50] was performed to make sure that those items with a high discrimination parameter (i.e., high slope) were retained in the IWPQ 1.0 [14,15]. Also, in the validation phase of the IWPQ scales, the IWPQ 1.0 was able to discriminate between known groups [20]. The fact that items with a high discrimination parameter were included in the IWPQ, and its good discriminant validity, suggest that it is likely that the IWPQ scales can also detect changes within groups over time.

Possibly, low responsiveness of the IWPQ could be caused by ceiling and floor effects in the scales. Although previous examination of the IWPQ using Rasch analysis has shown that the items of the IWPQ are relatively well-distributed over the scales, persons continue to score relatively high on task performance (ceiling effect), and low on CWB (floor effect); [15]. This could be caused by the tendency of persons to evaluate and present themselves in a socially desirable, favorable way [51,52]. As a consequence of the ceiling and floor effects, it becomes hard to detect further improvements in task performance, and further decreases in CWB. Thus, the ability to detect changes at the high part of the task performance scale, and low part of the CWB scale, may be diminished.

Another possible reason for the lower than expected correlations may lie in the study population. As said before, the population in the current study consisted of relatively healthy, well-functioning office workers who, in general, scored high on constructs such as general health, presenteeism, and job satisfaction, and low on constructs such as need for recovery, exhaustion, and sickness absenteeism. This makes it hard to obtain or detect any further improvements in this population. Despite the use of an intervention, small changes on the constructs over the 12-month intervention period were obtained. When examining the scatterplots of the change scores, low spread on many constructs can be observed (i.e., dots clustered in the middle), and this can cause deflated correlations [17].

Finally, a reason for the lower than expected correlations may be that the intervention was not effective enough to obtain changes in IWP. The primary aim of the Be Active & Relax study was to investigate the effectiveness of an intervention to stimulate physical activity and relaxation of office workers, on need for recovery [19]. Indirectly, an increase in physical activity and relaxation were expected to improve IWP. However, it may be that the intervention was not specific or intense enough to obtain improvements in IWP. Despite the fact that the intervention was not directly targeted at IWP, and despite high baseline levels on the constructs, a statistically significant increase in tasks performance (B = 0.2, 95% CI 0.0; 0.4), and a statistically significant decrease in contextual performance (B = -0.3, 95% CI -0.4; 0.1), were detected in the Be Active & Relax study [53]. The decrease in contextual performance could be explained by the fact that participants in the intervention groups were stimulated to engage in physical activity and relaxation during the workday, and this possibly could have reduced taking on extra work tasks, for example. Thus, this study showed that the IWPQ is able to detect statistically significant changes in individual work performance over time. Whether this change is a relevant change remains a question for future research.

### Assessment of responsiveness

As stated in the Introduction, there is a lot of confusion about the concept over responsiveness, and many different definitions and measures have been proposed over the past decades [17]. In addition, or perhaps, as a result, responsiveness is a seldom examined issue. For example, Abma et al. [54] reviewed the measurement properties of five self-report (health-related) work functioning instruments; the EWPS, WLQ, SPS, WPS, and LEAPS. For all five instruments, the methodological quality of responsiveness testing was poor, or not studied. Of the instruments used in the current study, only the responsiveness of the Need for Recovery Scale was examined. Based on effect sizes, the responsiveness of this scale appeared to be good [30]. However, the responsiveness of the other questionnaires used in the current study remains unknown. This is a limitation of the responsiveness testing process, because responsiveness of a new questionnaire is tested against change scores of existing questionnaires, whose responsiveness is also unknown, and may be poor.

No golden standard or clear guidelines seem to exist for the assessment of responsiveness and the interpretation of results. De Vet and colleagues [17] stated that responsiveness is often examined based on inappropriate outcome measures, such as effect sizes or standardized response mean. They advise that responsiveness should be seen as a form of longitudinal validity, using either a criterion approach (if a gold standard is available) or a construct approach (testing hypotheses of change scores).

In addition to the lack of clarity on how responsiveness should be tested, there are no clear guidelines as to what the strength of correlations between change scores should be. A final reason for the large percentage of unconfirmed hypotheses in the current study, may be that the hypothesized correlations (r = 0.30-0.50) were too high to begin with. In line with Cohen [37], we interpreted a correlation coefficient over 0.50 as strong, 0.30 to 0.50 as moderate, 0.10 to 0.30 as weak, and below 0.10 as no relation between constructs at all. Often, Cohen’s guidelines are used for cross-sectional correlations, i.e., when a correlation between two different measurement scores obtained at the same point in time is examined (thus, there is only one measurement). When it comes to correlations between change scores (multiple measurements), it is based on two measurements, and a double measurement error is involved. Due to this double measurement error, it seems reasonable that lower correlations may be expected. This issue has been addressed by other researchers. For example, Abma et al. [55] examined the responsiveness of the Work Role Functioning questionnaire, and they hypothesized correlation sizes around 0.20 to 0.30 with other constructs, because it was expected that many participants would show no changes, and based on results in earlier studies with similar questionnaires. For the constructs used in the current study, previous research has shown that, for example, the cross-sectional correlation between IWP and work engagement ranges between r = 0.30-0.50 e.g., [56]. It is therefore questionable whether correlations of r = 0.30-0.50 between their change scores can reasonably be expected. Such high correlations between change scores would likely be obtained for identical constructs, rather than similar (but not identical) constructs.

### Recommendations for future research

The current study provides merely a first step towards gaining insight into the responsiveness of the IWPQ. The responsiveness of the IWPQ should be further examined in future research, to determine whether its responsiveness is truly low, or whether the low responsiveness found in the current study was caused by limitations of the current study. We therefore recommend examining the responsiveness of the IWPQ in different populations, preferably in populations with low(er) baseline levels on the constructs, where large(r) changes on the constructs over time can be expected. Suggestions for such populations could be a sample of workers with work-related musculoskeletal health problems, mental health problems, and/or low job satisfaction. An intervention study, which is directly aimed at improving IWP, could obtain greater changes in these populations, making it easier to detect changes in IWP and related constructs. Suggestions for such a study could be an intervention focusing on managerial style, technological improvements at work, and/or job skills training. Also, the responsiveness of the IWPQ should preferably be examined using other measurement instruments of which the responsiveness is known. In addition, more information on the smallest detectable change and the minimally important change of the IWPQ, would further aid the interpretation of the responsiveness of the IWPQ. Finally, the responsiveness of questionnaires deserves greater attention, and clear guidelines for assessing and interpreting responsiveness should be adopted. The guidelines proposed by Terwee et al. [18], Mokkink et al. [57], and De Vet et al. [17] could provide a good starting point for this.

## Conclusion

Based on results of the current study, no firm conclusions can be drawn about the responsiveness of the IWPQ. Overall, most of the correlations between changes on the IWPQ scales and changes on other constructs were in the expected direction, although not as high as expected. This might indicate low responsiveness of the IWPQ. However, the weaker than expected correlations may also be accounted for by characteristics of the intervention study, such as the relatively healthy, well-functioning study population, and an intervention study that was not primarily aimed at IWP. Nevertheless, the IWPQ was able to show statistically significant changes in IWP during baseline and 12 months follow-up. Future research should provide more information about the smallest detectable change, the minimally important change, and the responsiveness of the IWPQ in other populations and intervention studies.

## Competing interests

The authors declare that they have no competing interests.

## Authors’ contributions

All authors have made substantial contributions to conception and design of the article. JKC and CRLB are responsible for data acquisition. LK is responsible for data analysis. All authors have contributed to interpretation of the data, and have been involved in drafting the manuscript or revising it critically for important intellectual content. All authors have given approval of the final version of the article, and agree to be accountable for all aspects of the work in ensuring that questions related to the accuracy or integrity of any part of the work are appropriately investigated and resolved.

## Pre-publication history

The pre-publication history for this paper can be accessed here:

http://www.biomedcentral.com/1471-2458/14/513/prepub
